# Bioconversion of Waste Fiber Sludge to Bacterial Nanocellulose and Use for Reinforcement of CTMP Paper Sheets

**DOI:** 10.3390/polym9090458

**Published:** 2017-09-18

**Authors:** Genqiang Chen, Guochao Wu, Björn Alriksson, Wei Wang, Feng F. Hong, Leif J. Jönsson

**Affiliations:** 1 College of Chemistry, Chemical Engineering and Biotechnology, Donghua University, Shanghai 201620, China; chengenqiang@gmail.com (G.C.); wangv@dhu.edu.cn (W.W.); 2 Department of Chemistry, Umeå University, Umeå SE-901 87, Sweden; guochao.wu@umu.se; 3 RISE Processum AB, SE-891 22 Örnsköldsvik, Sweden; bjorn.alriksson@processum.se

**Keywords:** bacterial cellulose, fiber sludge hydrolysate, stirred-tank reactor, chemithermomechanical pulp, paper sheet, tensile strength, tear resistance

## Abstract

Utilization of bacterial nanocellulose (BNC) for large-scale applications is restricted by low productivity in static cultures and by the high cost of the medium. Fiber sludge, a waste stream from pulp and paper mills, was enzymatically hydrolyzed to sugar, which was used for the production of BNC by the submerged cultivation of *Komagataeibacter xylinus*. Compared with a synthetic glucose-based medium, the productivity of purified BNC from the fiber sludge hydrolysate using shake-flasks was enhanced from 0.11 to 0.17 g/(L × d), although the average viscometric degree of polymerization (*DP*_v_) decreased from 6760 to 6050. The cultivation conditions used in stirred-tank reactors (STRs), including the stirring speed, the airflow, and the pH, were also investigated. Using STRs, the BNC productivity in fiber-sludge medium was increased to 0.32 g/(L × d) and the *DP*_v_ was increased to 6650. BNC produced from the fiber sludge hydrolysate was used as an additive in papermaking based on the chemithermomechanical pulp (CTMP) of birch. The introduction of BNC resulted in a significant enhancement of the mechanical strength of the paper sheets. With 10% (*w*/*w*) BNC in the CTMP/BNC mixture, the tear resistance was enhanced by 140%. SEM images showed that the BNC cross-linked and covered the surface of the CTMP fibers, resulting in enhanced mechanical strength.

## 1. Introduction

Bacterial nanocellulose (BNC) is mainly synthesized by acetic acid bacteria. As plant cellulose, BNC is an unbranched polymer composed of β-1,4-linked glucopyranose residues. However, BNC has much higher crystallinity and a higher degree of polymerization than plant cellulose [1,2]. The diameter of the BNC fiber, ranging from 20 to 80 nm, is smaller than that of other natural or synthetic nanofibers. BNC features many other important unique properties, including high purity, high wet tensile strength, a high Young's modulus, a large water-holding capacity, good shape maintenance, and excellent biocompatibility. These properties endow BNC with great potential in the areas of textile manufacturing, fiber-based paper and packaging products, food industry, biomedical materials, and advanced functional bionanocomposites [1,3,4]. However, these applications of BNC are restricted by its relatively high price. The high price is due to the high cost of the culture medium and the low productivity and yield of cellulose obtained in slow-growing static bacterial cultures [5,6,7].

To lower production costs, previous investigations have addressed production of BNC in static cultures from agro-industrial by-products and cellulosic residues such as konjak glucomannan [8], wheat straw [9,10], waste fiber sludge [11], spruce hydrolysate [12], and waste cotton textile [13,14]. Fiber sludge is a residual material obtained from pulp mills and biorefineries. It is usually relatively easy to hydrolyze fiber sludge enzymatically without prior thermochemical pretreatment, which is advantageous, as pretreatment usually results in formation of substances that inhibit the bacterium. Commonly-used bacterial strains are sensitive to well-known inhibitory compounds formed during pretreatment, except the aliphatic carboxylic acids [15,16]. Interestingly, it has been reported that fiber sludge hydrolysate could be used to produce BNC with the strain *Komagataeibacter xylinus* (formerly *Gluconacetobacter xylinus*) ATCC 23770 (American Type Culture Collection, Manassas, VA, USA) in static cultivation [11]. However, the possibility of submerged production of BNC from fiber sludge has not been examined.

To promote production of BNC, submerged cultivation in STRs (stirred-tank reactors) has been proposed as a possibility [17,18]. Compared with traditional static bacterial cultures, submerged cultivation in STRs would be less labor intense, allow higher glucose consumption rate and productivity, and facilitate scale-up. In the field of production of BNC in STRs, a few studies have been conducted with regard to cultivation conditions, such as medium pH, dissolved oxygen (DO), stirring speed, and concentration of culture medium components [19,20,21]. In addition, several other studies have been done on the structure of the STR; for example, comparisons of different impellers, and the introduction of a spin filter to the vessel [17,22]. However, these studies were focused on the productivity of BNC and rarely considered the effects of stirring on the structure and properties of the BNC, which would be significant if the BNC would be used for making materials. 

Previously, BNC has been employed as an additive in cotton lint pulp and kraft pulp of birch and pine, or as a coating material for print paper, or produced as special paper (such as a diaphragm of electroacoustic transducer and electronic paper). It was reported that BNC could enhance the mechanical strength and other paper properties (such as gloss value, trend to yellowing), though the detailed mechanism has not been studied [23,24,25,26,27,28,29,30]. Chemithermomechanical pulp (CTMP) is widely produced around the world, due to its high product yield in the manufacturing process. However, because of the relatively high lignin content in the CTMP, it is also characterized as being weak in mechanical strength, which means there is a demand for enhancement of the properties of CTMP-based paper products on the market. 

In this work, we addressed the problems of how to bio-convert waste fiber sludge into BNC in an STR and the benefits of including BNC in papermaking with CTMP. The study was started by employing a bacterial strain of *K. xylinus* that could produce BNC with high productivity and high *DP*_v_. Then, we investigated the possibility to produce BNC from fiber sludge hydrolysate, the effects of several parameters of cultivation in STRs on the BNC productivity and the *DP*_v_, and finally the effects of BNC on the mechanical properties of CTMP-based paper sheets.

## 2. Materials and Methods

### 2.1. Microorganism and Chemicals

A strain of *K. xylinus* (DHU-ZGD-1186) was kindly offered by Hainan Yeguo Foods Co., Ltd., Haikou, China, and was maintained in a glycerol stock at −80 °C. The glucose was of spectral grade. The peptone was from Merck KGaA (Darmstadt, Germany). The yeast extract was from VWR Chemicals (Radnor, PA, USA). Deionized water was used in all the experiments. The sulfite fiber sludge was cellulosic waste from the Domsjö Fabriker wood biorefinery in Örnsköldsvik, Sweden. Enzymatic hydrolysis of the fiber sludge was performed in a 75-L (working volume) bioreactor equipped with an anchor impeller (Gustav-Pilot Series, Belach Bioteknik, Stockholm, Sweden). Twenty kilograms dry-matter content (sulfite fiber sludge) and 10% (10 g/100 g dry sulfite fiber sludge) loading of a liquid enzyme preparation (Cellic CTec2, Novozymes, Bagsvaerd, Denmark) was used in a total hydrolysis reaction mixture of 50 kg. Enzymatic hydrolysis was performed at pH 5.0 and 50 °C, and using a stirring speed of 100 rpm. The duration of the hydrolysis was 40 h. After hydrolysis, the pH of the hydrolysate was adjusted to around 3 with sulfuric acid. The hydrolysate was then sterile-filtered and stored in a cold room. High-performance anion-exchange chromatography (HPAEC, Dionex ICS-3000 system, Sunnyvale, CA, USA) was used to determine the concentrations of monosaccharide sugars. The HPAEC system was equipped with a pulsed amperometric detector, a 3 mm × 30 mm guard column, and a 3 mm × 150 mm separation column (CarboPac PA20, Dionex). The sugar concentrations were 130.5 g/L glucose, 1.2 g/L xylose, and 0.4 g/L mannose. No other kinds of sugars were detected by using the HPAEC analysis. Before being used as culture medium, the fiber sludge hydrolysate was autoclaved at 105 °C for 1 h and then filtrated. The CTMP of birch was kindly offered by Dr. Mattias Andersson from Mid Sweden University (Örnsköldsvik, Sweden).

### 2.2. Culture Media

The seed culture medium contained 25 g/L glucose, 5 g/L peptone, and 3 g/L yeast extract. The pH was 5.0.

In a series of experiments on the feasibility of using fiber sludge hydrolysate as a carbon source for BNC production in shake-flask cultures, the fermentation culture medium contained 40 g/L glucose (which was either spectral grade glucose in reference fermentations or diluted fiber sludge hydrolysate), 10 g/L peptone, 6 g/L yeast extract, and 100 mM sodium acetate buffer. The final pH was adjusted to 5.0 using a 4 M aqueous solution of NaOH. The volume of medium in each 250-mL shake-flask was 100 mL. 

In a series of experiments on optimization of cultivation conditions in STRs, the fermentation culture medium contained 40 g/L glucose (from diluted fiber sludge hydrolysate), 10 g/L peptone, and 6 g/L yeast extract. The final pH was adjusted to 5.0 using the 4 M aqueous solution of NaOH. The volume of medium in each 400-mL STR was 300 mL.

### 2.3. Preparation of Seed Culture and Inoculation

One tube of glycerol stock of the bacterial strain was transferred to 100 mL seed culture medium, which was cultivated statically in an incubator at 30 °C for 4 days. Then, the inoculum was homogenized by using glass beads. The resulting homogenized seed culture was used for the inoculation (5% (*v*/*v*)) of both shake-flask and STR cultures. 

### 2.4. Study of Feasibility of Using Fiber Sludge Hydrolysate as a Carbon Source

Shake-flask cultivations were performed in an orbital shaker (Ecotron, Infors HT, Bottmingen Switzerland) set at 110 rpm and 30 °C. A sample of 1 mL culture fluid was taken every two days for determining pH and residual glucose concentration. After four and eight days of cultivation, 0.5 mL 10 M (pH 5.0) sodium acetate buffer was added to each shake-flask. BNC was harvested after 12 days. Triplicate cultivations were performed and mean values are reported.

### 2.5. Study on Optimization of Cultivation Conditions in STRs

The effect of stirring speed (50, 150, and 250 rpm) was studied at 30 °C and with an airflow of 1.0 vvm using a multibioreactor system (Multifors, Infors HT, Bottmingen, Switzerland) equipped with 400 mL vessels. The pH of the medium was not adjusted during the cultivation. The pH and DO of each culture were recorded every two days. The effect of airflow (0.5 and 1.5 vvm) was studied at 30 °C and a stirring speed of 250 rpm. The effect of adjustment of pH was studied at 30 °C using a stirring speed of 250 rpm and an airflow of 1.0 vvm. The pH of the medium in one bioreactor was not adjusted during the cultivation, while the pH of the medium of another bioreactor was adjusted to around 5 every two days using the 4 M aqueous solution of NaOH.

During cultivations, a sample of 1 mL culture broth was taken every two days for determining the concentration of residual glucose. After 12 days, crude BNC was harvested by centrifugation at 14,000× *g*. In order to assure that the purity of the BNC was sufficient for determining the average viscometric degree of polymerization (*DP*_v_), crude BNC was washed at least three times at 80 °C (each time for four hours) using a 0.1 M aqueous solution of NaOH, until the BNC became white. After that, it was washed at least five times with deionized water at 80 °C for four hours. The washing with NaOH solution was performed a larger number of times and for longer periods than which are commonly used (washing one time for no more than one hour) [19,31]. The purified BNC was freeze-dried and weighed. After washing and weighing, the *DP*_v_ was analyzed.

### 2.6. Analysis of Sugar Concentration

The glucose concentration was measured by using a glucometer (Accu-Chek Aviva, Roche Diabetes Care, Mannheim, Germany). A standard curve was established using a series of glucose solutions with known concentrations. Before measurements, broth samples were diluted 25 times with glucometer buffer (prepared as indicated by the manufacturer). The glucose concentration of each broth sample was measured three times, and the concentration values were recalculated using the standard curve. The concentrations of other monosaccharides were not measured, as they were negligible compared to glucose. 

### 2.7. Measurement of DP_v_ of BNC

The *DP*_v_ of the BNC was measured three times with a viscometer (Schott Gerate, Typ 52503/0c, K = 0.002879, Mainz, Germany) according to a method suggested by Evans and Wallis [32]. This method was modified from the American National Standard of ASTM D4243-99 [33], and is better for measuring the high *DP*_v_ of BNC according to Evans and Wallis [32]. 

### 2.8. Effects of BNC Addition on the Mechanical Properties of Birch CTMP Paper Sheets

CTMP and CTMP/BNC paper sheets were manufactured by MoRe Research AB (Örnsköldsvik, Sweden), who also determined the mechanical properties. Purified BNC from the optimal cultivation conditions was disintegrated using a blender (YD-2318S, Clas Ohlson, Insjön, Sweden) at a concentration of around 2 g/L for 6 min. The exact dry mass of the BNC in the homogenate was measured. Laboratory wet disintegration of the CTMP of birch was performed according to ISO 5263-3:2004 [34]. The homogenate of BNC was mixed with the disintegrated CTMP to achieve concentrations of 5% and 10% (*w*/*w*) BNC in the CTMP/BNC mixtures. The mixture was mixed using an impeller at 1500 rpm for 5 min. Paper sheets were made from the mixture according to the Rapid–Köthen method (ISO 5269-2:2004) [35]. A paper filter with a pore size of 7–10 µm (242001, Munktell, Falun, Sweden) and a polyester filter with a pore size of around 71 µm (PES71N, Momodur, Eczacıbaşı Anasayfa, Istanbul, Turkey) were put on the sheet-forming screen of the Rapid–Köthen apparatus to enhance the retention of BNC. All tests were made in a climate room, as specified in ISO 187:1990 [36]. The grammage of the paper sheets was measured according to ISO 536:2012 [37]. The tensile strength of the paper sheets was determined according to ISO 1924-3:2005 [38]. The tear resistance was studied based on ISO 1974:2012 [39].

### 2.9. Scanning Electron Microscopy

The paper samples were placed onto a carbon tape-mounted aluminum stub, and sputtered-coated with 5 nm Au/Pd (Quorum Q150T ES, Lewes, United Kingdom). The morphology of the samples was examined by using field-emission scanning electron microscopy (FESEM) (Merlin, Carl Zeiss AG, Jena, Germany) equipped with an in-lens secondary electron detector at accelerating voltage of 4 kV and a probe current of 120 pA.

## 3. Results and Discussion

### 3.1. Feasibility of Using Fiber Sludge Hydrolysate as a Carbon Source in Shake-Flask Cultures

**Figure 1:**
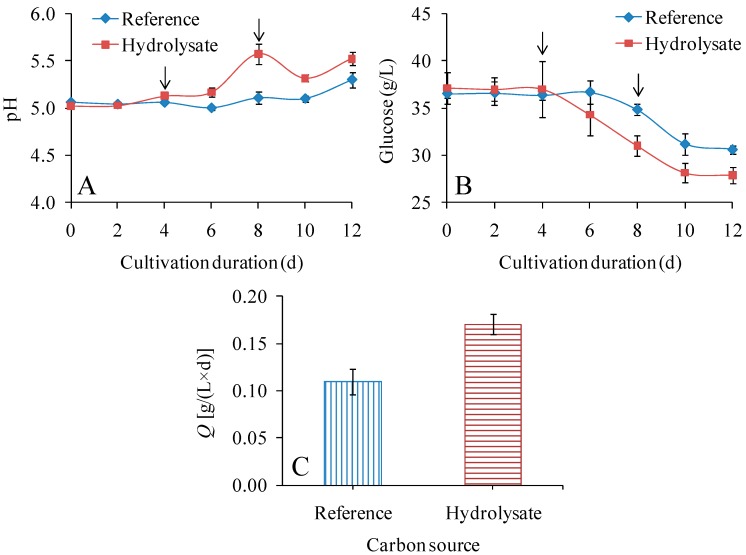
Graphs showing pH (**A**), residual glucose concentration (**B**), and volumetric productivity (**C**) in reference medium and hydrolysate-based medium in shake-flask experiments. The arrows show time points for addition of acetate buffer during the cultivations. The volume of the culture medium was 100 mL (250-mL flasks), the shaking speed was 110 rpm, and the cultivation temperature was 30 °C.

In the first series of experiments, the fiber sludge hydrolysate was compared with a reference medium based on synthetic glucose with regard to fermentability (measured as glucose consumption) and the productivity, yield, and *DP*_v_ of BNC. As acetate buffer could increase production of BNC [40], the media were buffered with sodium acetate. The pH adjustments with sodium acetate were carried out on days 4 and 8 (Figure 1A). However, there was a lag phase of four days for both media, and even at the end of the cultivation much of the glucose was still left (Figure 1B). The glucose consumption in the hydrolysate was faster than in the reference medium (Figure 1B, Table 1), but still no more than 0.77 [g/(L × d)]. As the overall glucose consumption (Figure 1B) and the BNC yield on initial glucose (0.036–0.055 g/g, Table 1) were rather modest, the following experimental series were carried out without acetate buffer and mostly without pH adjustment.

The value for volumetric productivity of BNC (Table 1) was 55% higher for the hydrolysate medium than for the reference medium. Similarly, the value for BNC yield on initial sugar (Table 1) was 53% higher for the hydrolysate medium than for the reference medium. The yields on consumed glucose were, however, quite similar: 0.235 g/g for the reference medium and 0.223 g/g for the hydrolysate (Table 1). It is possible that denatured cellulase and other trace components in the hydrolysate promoted bacterial metabolism and growth. As a result, glucose consumption and especially BNC production might have been enhanced. The *DP*_v_ was slightly higher for the BNC from the reference medium (6760) than for the BNC from the hydrolysate medium (6050) (Table 1).

Compared with many other studies in the field, the volumetric BNC productivity, the yield of BNC, and the *DP*_v_ were generally good, despite using submerged cultivations, a medium based on industrial residues, and a very extensive washing scheme. Cavka et al. [11] investigated the production of BNC from enzymatic hydrolysate of waste fiber sludge by *K. xylinus* ATCC 23770 after static cultivation. Using hydrolysates of sulfite fiber sludge as carbon source, they found that volumetric productivity of BNC was 5% lower than for a medium based on synthetic glucose [11], while in the current study it was instead >50% higher. The advantageous effect on BNC productivity observed in our study could possibly be related to better mixing in submerged cultivation and to the special characteristics of strain DHU-ZGD-1186. Using static cultivation and 200 mM acetate buffer in the medium, Kuo et al. [40] reached a BNC yield on consumed glucose of 0.23 g/g, which can be compared to 0.22–0.24 g/g in our study. In the study of Shibazaki et al. [41], *K. xylinus* ATCC 23769 was cultivated for 5–7 days, and then the BNC was harvested and purified by boiling in a 1% solution of NaOH. Measuring DP with a similar method [42] as we used in our study, they reported a *DP*_v_ of 2000 for BNC and 2280 for cotton linter [41]. This can be compared with the *DP*_v_ values of the BNC in our study, which were always >6000. The higher *DP*_v_ of the BNC in our study could possibly be attributed to the characteristics of strain DHU-ZGD-1186, to the longer duration of the cultivation (12 days), and to the lower NaOH concentration (4 g/L) in the washing.

### 3.2. Effect of Stirring Speed on the Production of BNC in STRs

As the results achieved in shake-flasks indicated that the hydrolysate was about as good as or even better than the glucose-based reference medium, the next step was moving from shake-flasks to STRs for improving submerged cultivation conditions for fiber sludge hydrolysate. The stirring speed, the aeration, and the potential benefits of pH adjustment were studied. 

**Figure 2:**
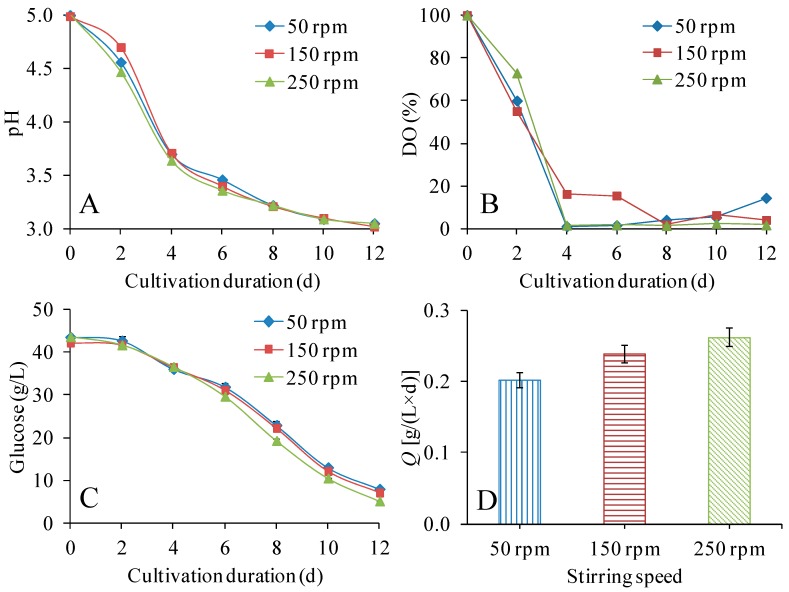
Effects of stirring speed on pH (**A**), dissolved oxygen (DO) (**B**), residual glucose concentration (**C**), and volumetric productivity (**D**). The volume of culture medium was 300 mL (in 400 mL stirred-tank reactors (STRs)), and the cultivation temperature was 30 °C.

In the first series of bioreactor experiments, stirring speeds in the range 50–250 rpm were compared (Figure 2, Table 2). As the medium was not buffered, the pH dropped from 5 to about 3 during the cultivation (Figure 2A). The change in pH was the same for all three stirring speeds tested, and there was no sign of a four-day lag phase as in the shake-flask experiment (Figure 2A). Instead, the pH dropped fast from 5 to about 3.5 during the first four days, and after that it decreased slowly to about 3 during the next eight days (Figure 2A). Similarly, the DO dropped rapidly during the first four days and remained at low levels until the end of the cultivation (Figure 2B). 

Even if glucose consumption was slow for the first two days, the data (Figure 2C) indicated a clear improvement compared to the shake-flask experiments (Figure 1B). That could be attributed to better mixing of the medium and better air supply. The improvement compared to the shake-flask cultures is further highlighted by data in Table 2, which shows glucose consumption rates of about 3 g/(L × d) and volumetric BNC productivities ranging from 0.20 to 0.26 g/(L × d). Whereas no clear difference in glucose consumption for 50 and 150 rpm was detected (Figure 2, Table 2), the volumetric BNC productivity and the BNC yield on initial glucose steadily increased with increasing stirring speed (Table 2). The BNC yield of consumed glucose was in the range 0.068–0.082 g/g, significantly lower than in the shake-flask experiments and independently of the medium with which the comparison was made (Table 1). The decrease in yield on consumed glucose may be related to the absence of acetate buffer in the STRs as compared with the shake-flasks. Kuo et al. [40] found that the yield of BNC on consumed glucose in medium without acetate buffer was just 0.06 g/g, much lower than the 0.23 g/g achieved in medium with 200 mM acetate buffer. They also found higher levels of the by-product gluconate (15.02 g/L) in the medium without acetate buffer than in the medium with acetate buffer (11.46 g/L) [40], which would have contributed to lower BNC yield on consumed glucose. 

The *DP*_v_ values were in the narrow range 6210–6480, with the highest stirring speed giving the lowest *DP*_v_ (Table 2). Comparing the same medium, the *DP*_v_ values in the bioreactor experiment (Table 2) were higher than the *DP*_v_ value in the shake-flask experiment (Table 1). The difference between 6390 (50 rpm) and 6480 (150 rpm) was not significant (*p* > 0.05), but the decrease from 6480 to 6210 was significant (*p* ≤ 0.01). Watanabe et al. [31] reported that agitation could result in decreased *DP*_v_ of BNC. They produced BNC in a 1 L STR with stirring speed high enough to maintain the DO concentration above 1.0 ppm. The weight-average degree of polymerization of the BNC from the STR cultivation was found to be 10,900, lower than that of 14,400 from static cultivation [31]. 

### 3.3. Effect of Airflow on the Production of BNC in STRs

**Figure 3:**
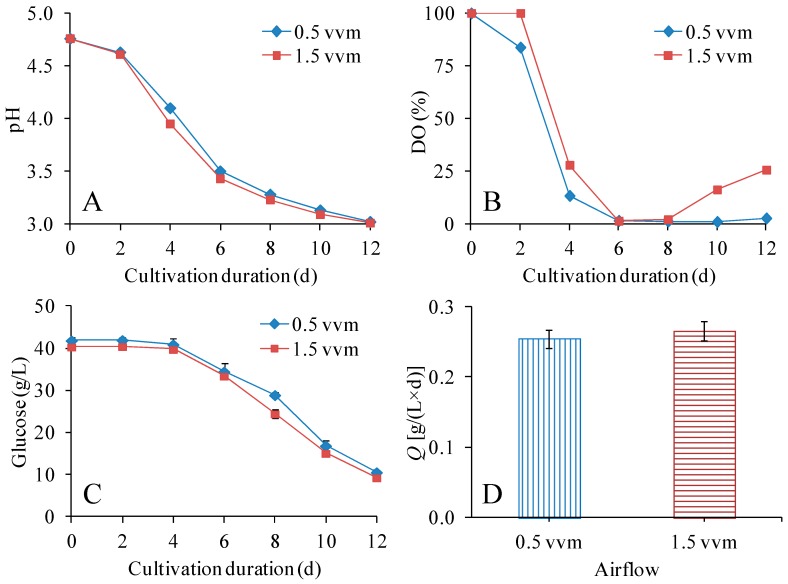
Effects of airflow on pH (**A**), DO (**B**), residual glucose concentration (**C**), and volumetric productivity (**D**). The volume of the culture medium was 300 mL (400-mL STR) and the cultivation temperature was 30 °C.

In the experimental series with different stirring speeds, the air flow was set to 1 vvm; in the second set of experiments in the bioreactor, this setting was compared to lower (0.5 vvm) and higher (1.5 vvm) airflow, using 250 rpm as the stirring speed (Figure 3, Table 2). As seen in Figure 3A,C, the pH change and the glucose consumption were similar in bioreactors with different airflow. The glucose consumption rate was about 2.6 g/(L × d) (Table 2) with no significant (*p* > 0.05) differences. As seen in Figure 3B, the DO was higher when the airflow was 1.5 vvm, especially at the beginning and at the end of the cultivation. 

The values for volumetric productivity of BNC, BNC yield on initial glucose, and BNC yield on consumed glucose were higher for cultivation with 1.5 vvm than for cultivation with 0.5 vvm, but the differences were not significant (*p* > 0.05). Although that effect could be seen in our experiments (Table 2), it has been reported that increasing the airflow could increase the productivity of BNC, due to the decreased partial pressure of CO_2_ in the gaseous phase [21]. 

With an airflow of 0.5 and 1.5 vvm, the *DP*_v_ of the BNC was 6650 and 6200, respectively (Table 2). It is speculated that the high airflow of 1.5 vvm perhaps disturbed the biosynthesis of BNC, especially before the BNC turned to gel. The *DP*_v_ with 1.5 vvm was similar to the *DP*_v_ with 1.0 vvm (6210) (Table 2). It is noteworthy that while both high stirring speed and high air flow tended to result in lower *DP*_v_, a high *DP*_v_ (6650) could still be achieved by combining high stirring speed (250 rpm) with low air flow (0.5 vvm) without any significant decreases in volumetric productivity of BNC, BNC yield on initial glucose, or BNC yield on consumed glucose (Table 2).

### 3.4. Effect of pH Adjustment on the Production of BNC in STRs

**Figure 4:**
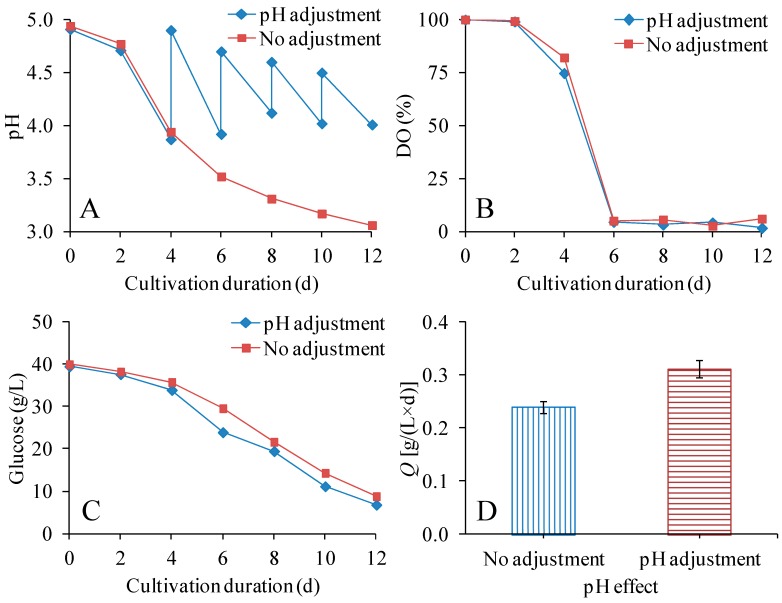
Effects of pH adjustment on pH (**A**), DO (**B**), residual glucose concentration (**C**), and volumetric productivity (**D**). The volume of culture medium was 300 mL (400-mL STR) and the cultivation temperature was 30 °C.

Cultivation in acetate-buffered medium in shake-flasks resulted in relatively low sugar utilization, and most of the experiments in STRs were performed without pH control; however, one experimental series covered pH adjustment in STRs using a solution of sodium hydroxide (Figure 4, Table 2). Figure 4A shows the pH during the cultivations, which were performed using a stirring speed of 250 rpm and an air flow of 1.0 vvm. Without pH adjustment, the pH decreased to about 3.0 after cultivation for 12 days. With pH adjustment, the pH was kept within the range 4.0–5.0 (Figure 4A). 

The effects of pH adjustment on DO (Figure 4B) and glucose consumption (Figure 4C) were rather small. However, the pH adjustment had a clear positive effect on volumetric productivity of BNC, BNC yield on initial glucose, and BNC yield on consumed glucose (Table 2). This result agrees with reports that the pH of the medium strongly affects BNC production and that the optimal pH for BNC production is in the range of 4.0–6.0, as this is a favorable pH range for the bacteria [20,43]. An adjustment of pH resulted in a decrease of the *DP*_v_ of BNC from 6310 to 6080 (Table 2). This could possibly be related to the function of the cellulase produced by *K. xylinus*. The pH range of 4.0–5.0 in the medium with pH adjustment could lead to higher cellulase activity and result in lower *DP*_v_. Previously Tahara et al. [44] studied the pH effect on BNC productivity and *DP*_v_. They found that in a medium with a constant pH of 5, both the BNC productivity and the cellulase activity were higher than in a medium with pH 4. Due to the higher cellulase activity, the *DP*_v_ decreased in the medium with a pH of 5 [44].

### 3.5. Effects of BNC Addition on the Mechanical Properties of Birch CTMP Paper Sheets

A series of CTMP paper sheets containing no BNC, 5% BNC, and 10% BNC was prepared to test the effects of inclusion of BNC on the mechanical properties of the paper sheets, which are shown in Table 3. The addition of 5% or 10% BNC did not have any clear effect on grammage or thickness, but a slight increase in density could be discerned (Table 3). The addition of BNC did, however, result in increases of tensile index, tensile stiffness index, tensile energy absorption index, strain at break, E-modulus, and tear index (Table 3). For example, the increase in tensile index amounted to 16% for 5% BNC and 49% for 10% BNC. The increase in tear index amounted to 66% for 5% BNC and 140% for 10% BNC. In most cases, there were significant differences not only between BNC-less and BNC-containing paper sheets (*p* ≤ 0.05), but also between paper sheets containing 5% and 10% BNC (*p* ≤ 0.05). An exception was the strain at break, for which there was no significant difference between sheets with 0%, 5% or 10% BNC (*p* > 0.05) (Table 3). 

Our results show that introduction of BNC could significantly enhance the mechanical properties of CTMP-based paper sheets. It has been reported that BNC could enhance the tensile properties and tear index of paper made of cotton lint pulp, softwood pulp, and kraft pulp of birch and pine [23,24,45]. Surma–Ślusarska et al. found that, with 15% disintegrated BNC in paper sheets of kraft pulp of birch, the tear index was enhanced by 33% [45]. This can be compared with our results showing an increase in tear index by 66% for paper sheets with 5% BNC and an increase with 140% for paper sheets with 10% BNC. The higher increase observed in our study can be explained by the use of different types of pulp and different paper qualities, but possibly also by the use of BNC of different qualities, such as different DP.

**Figure 5:**
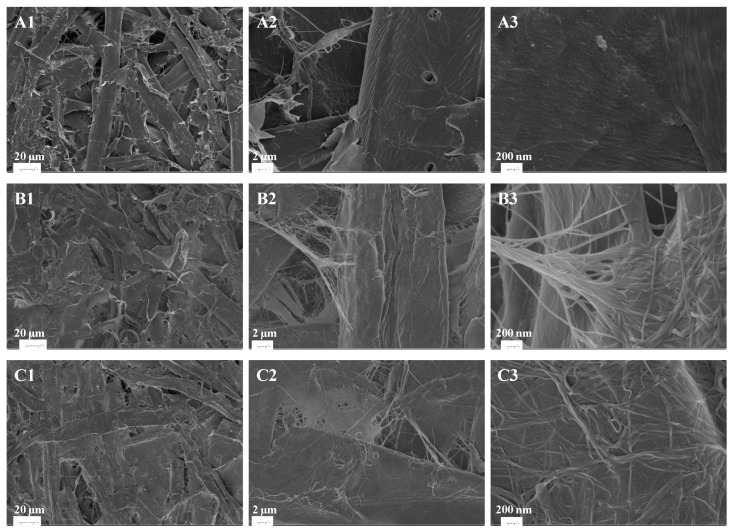
SEM images of paper sheets of birch CTMP, and CTMP and BNC mixtures. Fraction of BNC: (**A1**–**A3**) 0 *w*/*w* %; (**B1**–**B3**) 5 *w*/*w* %; (**C1**–**C3**) 10 *w*/*w* %. Amplifications: (**A1**–**C1**) 750×; (**A2**–**C2**) 5000×; (**A3**–**C3**) 50,000×.

To explain the mechanism of the enhancement in more detail, SEM images of the paper sheets were taken, as shown in Figure 5. Figure 5(B1) and 5(C1) (magnification 750×) show that the BNC covered the gap between CTMP fibers. Figure 5(B2) and 5(C2) (magnification 5000×) show that the BNC cross-linked different CTMP fibers, and that it also covered the surface of the CTMP fibers, which is also shown in Figure 5(B3) and 5(C3) (magnification 50,000×). Figure 5(B3) and 5(C3) show particularly well how single BNC fibers were in contact with the pulp fibers. Since the BNC attached strongly to the surface of the CTMP fibers, the CTMP fibers became difficult to separate from each other, resulting in a great improvement of the tear index. 

## 4. Conclusions

This work shows how an enzymic hydrolysate of waste fiber sludge could be converted to high value-added BNC and be used to enhance the properties of CTMP paper sheets, thus taking care of a waste stream and enhancing a product, both of which come from the forest industry. The data indicate that the fiber sludge hydrolysate was even better than a reference medium based on a synthetic glucose solution. The investigation also shows important connections between cultivation conditions and the yield and DP of the BNC; for instance, regarding submerged cultivation in shake-flasks vs. cultivation in STRs, stirring speed, aeration, and pH adjustment. Initial studies of the effects of BNC on papermaking using CTMP were also performed, and it was shown that the BNC could significantly enhance the mechanical properties of the paper and, especially, the tear resistance, as BNC could cross-link the CTMP fibers and cover their surface. More investigations are needed to clarify why fiber sludge hydrolysates can be superior to synthetic glucose for BNC production, to decrease the enzyme cost and other costs, to up-scale BNC production in STRs, and to further explore and optimize the use of BNC to reinforce materials based on plant cellulose.

## Figures and Tables

**Table 1:** Glucose consumption, bacterial nanocellulose (BNC) productivity, BNC yield, and viscometric degree of polymerization (*DP*_v_) in shake-flask experiments ^a^.

Medium	Glucose Consumption Rate [g/(L × d)]	*Q* [g/(L × d)]	*Y*_P/initial G_ (g/g)	*Y*_P/consumed G_ (g/g)	*DP*_v_
Reference	0.47 ± 0.04	0.110 ± 0.013	0.036 ± 0.003	0.235 ± 0.010	6760 ± 110
Hydrolysate	0.77 ± 0.07	0.171 ± 0.011	0.055 ± 0.001	0.223 ± 0.010	6050 ± 80

^a^ The BNC was purified by washing with alkali and water.

**Table 2:** Influence of stirring speed, airflow and pH adjustment of the fiber sludge hydrolysate medium on glucose consumption, BNC productivity, BNC yield, and *DP*_v_. The experiments with different variables were conducted separately ^a^.

Variable	Conditions	Glucose Consumption Rate [g/(L × d)]	*Q* [g/(L × d)]	*Y*_P/initial G_ (g/g)	*Y*_P/consumed G_ (g/g)	*DP*_v_
Stirring speed (1.0 vvm)	50 rpm	2.96 ± 0.07	0.202 ± 0.010	0.056 ± 0.002	0.068 ± 0.006	6390 ± 90
	150 rpm	2.93 ± 0.05	0.239 ± 0.011	0.068 ± 0.002	0.082 ± 0.003	6480 ± 70
	250 rpm	3.21 ± 0.04	0.262 ± 0.013	0.072 ± 0.002	0.082 ± 0.003	6210 ± 30
Airflow (250 rpm)	0.5 vvm	2.61 ± 0.11	0.255 ± 0.013	0.073 ± 0.004	0.098 ± 0.007	6650 ± 70
	1.5 vvm	2.58 ± 0.04	0.266 ± 0.013	0.079 ± 0.004	0.103 ± 0.007	6200 ± 60
pH adjustment (250 rpm, 1.0 vvm)	pH adjustment	2.72 ± 0.03	0.312 ± 0.016	0.095 ± 0.005	0.115 ± 0.006	6080 ± 50
	No adjustment	2.59 ± 0.03	0.240 ± 0.012	0.072 ± 0.004	0.092 ± 0.005	6310 ± 90

^a^ The BNC was purified by washing with alkali and water.

**Table 3:** Mechanical properties of paper sheets made from chemithermomechanical pulp (CTMP) with BNC as an additive.

Fraction of BNC (*w*/*w* %)	0	5	10
Grammage, paper (g/m^2^)	57.1 ± 2.6	59.0 ± 1.4	58.7 ± 3.2
Thickness (mm)	0.16 ± 0.01	0.16 ± 0.01	0.15 ± 0.01
Density (kg/m^3^)	354 ± 20	374 ± 7	389 ± 8
Tensile Index (kNm/kg) ^a^	16.6 ± 1.7	19.3 ± 1.5	24.7 ± 2.5
Tensile stiffness index (MNm/kg) ^b^	3.45 ± 0.30	4.05 ± 0.11	4.97 ± 0.43
Tensile energy absorption index (J/kg) ^c^	56.7 ± 9.2	69.3 ± 10.9	87.7 ± 15.7
Strain at break (%)	0.601 ± 0.032	0.624 ± 0.061	0.626 ± 0.075
E-modulus (GPa)	1.22 ± 0.08	1.51 ± 0.05	1.89 ± 0.20
Tear Index (mNm^2^/g) ^d^	1.35 ± 0.28	2.24 ± 0.07	3.20 ± 0.20

^a^ Tensile strength determined as the maximum tensile force per unit width the sheet will withstand before breaking, and tensile index determined as tensile strength divided by grammage. ^b^ The tensile stiffness determined as the maximum slope of the curve obtained when tensile force per unit width is plotted versus strain, and tensile stiffness index determined as tensile stiffness divided by grammage. Elastic modulus (E-modulus) determined as tensile stiffness divided by thickness. ^c^ Tensile energy absorption determined as the amount of energy per unit surface area (test length × width) of a test piece when it is strained to the maximum tensile force. Tensile energy absorption index determined as tensile energy absorption divided by grammage. ^d^ Tearing resistance determined as the average force per sheet required to continue the tearing started by an initial cut in the test piece. Tearing index determined as tearing resistance divided by grammage.
